# Medicinal Plants Utilized for the Treatment of Gastrointestinal Parasitosis in Ethiopia

**DOI:** 10.1155/2022/3584861

**Published:** 2022-03-16

**Authors:** Yibeltal Aschale, Haimanot Reta, Awoke Minwuyelet, Animen Ayehu, Muluken Wubetu

**Affiliations:** ^1^Department of Medical Laboratory Science, College of Health Sciences, Debre Markos University, Debre Markos, Ethiopia; ^2^Department of Biology, College of Natural and Computational Science, Debre Markos University, Debre Markos, Ethiopia; ^3^Medical Parasitologist, Bichena Primary Hospital, Amhara Region, Ethiopia; ^4^Department of Medical Laboratory Sciences, College of Medicine and Health Sciences, Bahir Dar University, Bahir Dar, Ethiopia; ^5^Department of Pharmacy, College of Health Sciences, Debre Markos University, Debre Markos, Ethiopia

## Abstract

**Background:**

Nonhygienic living conditions give rise to parasitic infections. Intestinal parasitosis is a serious public health problem in Ethiopia. It is more common in the poor part of the population with low-income, poor personal, and environmental sanitation and limited clean water supply. This review is aimed at providing an overview of the medicinal plants used for the treatment of gastrointestinal parasitosis in Ethiopia.

**Methods:**

International databases (PubMed, Google Scholar, Scopus, and Web of Science) were systematically searched to access published original articles on medicinal plants used to treat gastrointestinal parasitosis without restriction on the year of publication and methodology. The validity of articles was checked before inclusion in the review by undertaking critical appraisal using tools adapted from JBI Critical Appraisal Checklist. The details of medicinal plants were extracted from the included studies using a standardized data extraction format in excel spreadsheet and analyzed using descriptive statistics to calculate frequency and percentage.

**Results:**

A search for published articles produced a total of 205 papers, of which 23 met the inclusion criteria. Of the 85 medicinal plants identified, the majority (40.2%) were shrubs, and the common plant part used was leaf (28.2%). Family Asteraceae has the highest number of plant species. The majority of the plant remedies were given orally (96.9%). Taeniasis comprises the highest percentage of intestinal parasitosis treated followed by ascariasis.

**Conclusion:**

Numerous plants have been utilized to treat gastrointestinal parasitosis. Information obtained from this review could serve as a guide to discover novel antiparasitic agents. Therefore, it is advisable for researchers to properly identify, document, conserve, and conduct safety and efficacy studies on such claimed medicinal plants.

## 1. Background

Traditional medicine has been adopted in Ethiopia since a long time ago along with scientific treatment modalities. Even nowadays, plants have been used extensively to treat various human and animal health problems [1]. Many end products with efficient antiparasitic activities are derived from plants [2]. These end products have significant contributions to human and animal illnesses for the development of new drugs, because the compounds found in plants contain an abundant source of active compounds that can be a novel antiparasitic agent [3, 4]. It is estimated that about 80% of the population in Ethiopia still uses medicinal plants to treat a variety of health problems. Not only Ethiopians but also around 60% of the world population depends on traditional medicine [5].

Intestinal parasitosis is the most common infection in developing countries including Ethiopia. A high burden is documented among economically poor sections of the population; preschool and school children were the most affected groups [6]. Poor personal and environmental hygiene, eating uncooked or undercooked food/meat, close contact with domestic animals, lack of safe water supply, and hot climate are some of the factors associated with intestinal parasite infections. Amoebiasis and ascariasis are the leading infections that affect the gastrointestinal tract among the ten top intestinal parasitic infections globally [7]. They are recognized as serious public health problems causing iron-deficiency anemia, malnutrition, diarrheal illness, growth, and mental retardation largely in children [8].

Despite the great role of traditional medicine in primary health care, little work has so far been done in Ethiopia to properly identify, document, and conserve medicinal plants and associated data. There is a need to provide full ethnobotanical data on traditionally claimed medicinal plants used to treat gastrointestinal parasitosis. Therefore, this review is aimed at broadly identifying medicinal plants used for the treatment of gastrointestinal parasitosis in Ethiopia.

## 2. Materials and Methods

### 2.1. Search Strategy

International electronic databases (PubMed, Google Scholar, Scopus, and Web of Science) were systematically searched for published articles about medicinal plants utilized for the treatment of gastrointestinal parasitosis in Ethiopia. All searches were limited to articles published in English. This review has used the Preferred Reporting of Systematic Reviews and Meta-Analysis (PRISMA) guideline to assure scientific rigor. The search was conducted without restriction on the year of publication and methodology. Common search terms were “intestinal parasites,” “Ethiopia,” “medicinal plants,” and “ethnomedicine.” The search terms were used separately and in combination using Boolean operators like “OR” or “AND.” The reference lists of included studies were screened for additional eligible studies.

### 2.2. Article Selection Criteria

Original studies which contain full ethnobotanical information (family name, scientific name, growth form, plant parts used, specific use, and route of administration) were included in the review. Studies that incorporate only medicinal plants for livestock usage were excluded. Plants that are out of the flora list of Ethiopia and Eritrea were excluded from the review [9].

### 2.3. Assessment of Methodological Quality

The methodological validity of all 23 studies was checked before inclusion in the review by two investigators by undertaking critical appraisal using standardized tools adapted from JBI Critical Appraisal Checklist [10] to incorporate into the review process. If disagreements between two assessors occur it was resolved by discussion and by contacting the primary author of the included studies via email.

### 2.4. Data Extraction

The details of medicinal plants (scientific, family, and local names, growth forms of plants, plant parts used, methods of preparation, specific use, and route of administration) were extracted from included studies using a standardized data extraction form adapted from the JBI data extraction format. All required information on medicinal plants was extracted and recorded in an excel spreadsheet. The collected data were analyzed using descriptive statistics to calculate frequency and percentage.

## 3. Results

**Figure 1:**
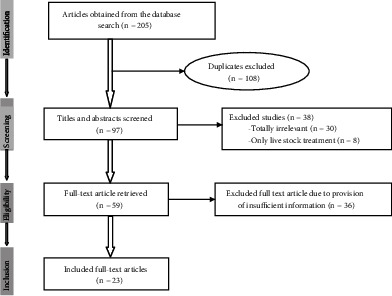
Flowchart of study selection for review of medicinal plants utilized for the treatment of gastrointestinal parasitosis in Ethiopia.

A total of 205 original articles were obtained from the database search. After removing duplicates and screening of titles, abstracts, and full contents, 23 studies met the inclusion criteria and were found suitable for the review (Figure 1). In this ethnobotanical review, 85 medicinal plants (distributed in 46 families) were identified and found application by the traditional healers to treat gastrointestinal intestinal parasitosis. The detailed description of each medicinal plant recorded from the included studies is available in Table S1 [11–33].

### 3.1. Frequently Used Families

**Figure 2:**
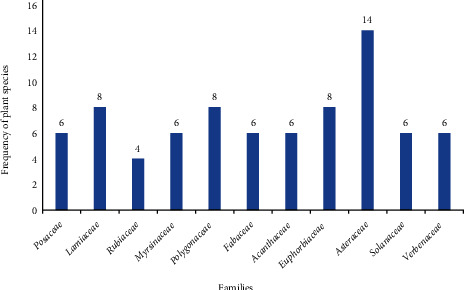
Frequently used families for the treatment of gastrointestinal parasitosis.

Families *Asteraceae*, *Rosaceae*, *Solanaceae*, *Euphorbiaceae*, *Polygonaceae*, and *Lamiaceae* were some of the commonly utilized plant families (Figure 2).

### 3.2. Frequently Used Plant Species

**Figure 3:**
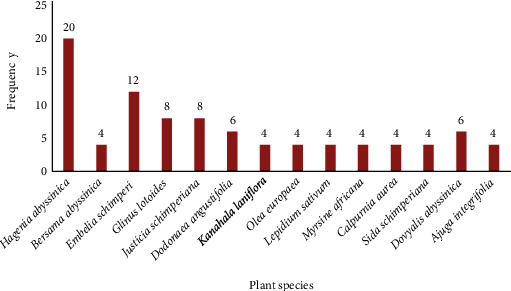
Frequently used plant species for the treatment of gastrointestinal parasitosis.

The most frequently used plant species were *Hagenia abyssinica*, *Embelia schimperi*, *Justicia schimperiana*, *Dodonaea Angustifolia*, and *Glinus lotoides* (Figure 3).

### 3.3. Solvents and Additives Used

**Figure 4:**
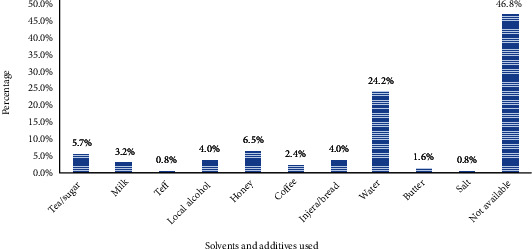
Solvents and additives used by healers for the preparation of remedy in Ethiopia.

Traditional healers used water, honey, tea, coffee, and milk as a solvent or additive for their remedy preparation (Figure 4).

### 3.4. Growth Forms of Medicinal Plants and Plant Parts Used

**Figure 5:**
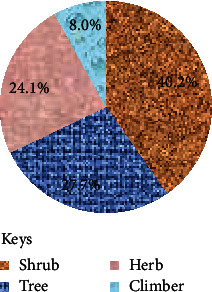
Growth forms of medicinal plants used for the treatment of intestinal parasitosis.

**Figure 6:**
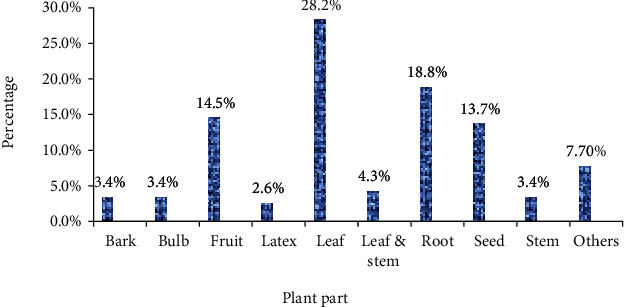
Plant parts used for the treatment of intestinal parasitosis in Ethiopia.

Of the medicinal plants identified, the majority of them were shrubs (40.2%), followed by trees (27.7%), herbs (24.1%), and climbers (8.0%) (Figure 5). The commonly used plant parts were leaves (28.2%) followed by roots (18.8%) (Figure 6).

### 3.5. Method of Preparation and Route of Administration

**Figure 7:**
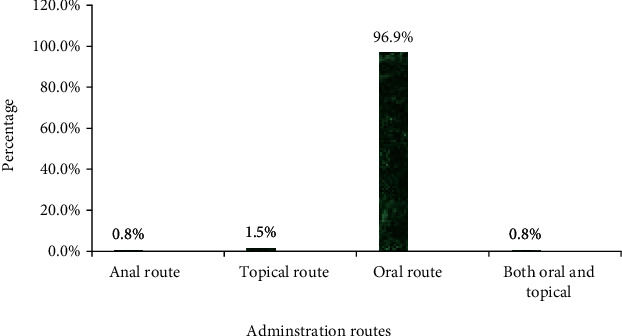
Route of administration of medicinal plants used for the treatment of intestinal parasitosis.

Traditional medicinal practitioners use simple compounding procedures like drying, crushing, concoction, and decoction (Table S1). They prefer the oral route as the best administration route (96.9%). Other routes were also used to give remedies (topical = 1.5%, anal = 0.8%, combination of oral and topical = 0.8%) (Figure 7).

### 3.6. Intestinal Parasite Infections Treated with Medicinal Plants

**Figure 8:**
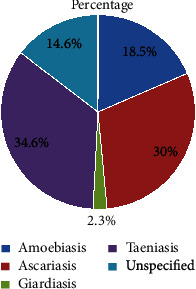
Proportion of intestinal parasite infections treated by medicinal plants in Ethiopia.

Taeniasis, ascariasis, amoebiasis, and giardiasis were the most common intestinal parasite infections that are treated traditionally when encountered in humans. Taeniasis accounts 34.6%, followed by ascariasis (30%), amoebiasis (18.5%), and giardiasis (2.3%) (Figure 8).

## 4. Discussion

Ethnobotany is a useful approach for pharmaceutical research and novel drug discovery. Claimed medicinal plants play a great role in this purpose. This review identified 85 medicinal plant species having been applied by traditional healers to treat gastrointestinal parasitosis. This indicates the extent of knowledge present in different people of Ethiopia having diverse cultures. High species diversity of medicinal plants is observed which might be due to the climate variation that exists in the country. Family Asteraceae has the highest number of plant species. The activity of this family against gastrointestinal parasites might be due to the presence of bioactive phytochemicals such as steroids, flavonoids, phenolic acids, glycosides, triterpinoids, and esters that have strong antioxidant, anticholinesterase, and scavenging activities [34]. In glycosides, level of activity is dependent on the nature of the sugar side-chain at the C-3 position [35]. Although there are claims on antihelminthic, antiamoeba, and antigiardial activity in Asteraceae, the bioactivity properties of phytochemicals against these parasites is not well studied. Moreover, phytochemical screening studies showed that herbal products from this family may cure gastrointestinal disorders by treating diarrhea and dysentery. This study is supported by studies done elsewhere [36, 37]. However, a study conducted in the Hawassa Zuria district showed the dominance of the family Fabaceae which might be due to climatic variations [38].

According to this review, most plants belong to shrubs followed by trees and herbs. However, other studies conducted elsewhere in Ethiopia indicated the dominance of herbs [24, 38–42]. Extensive use of shrubs in the preparation of remedies might be due to easy accessibility in the country. Leaf was found to be the most commonly used plant part followed by root. This was in agreement with other research works [38, 43, 44]. The use of the leaf part of the plant frequently might be due to easy formulation into different dosage forms. However, extensive use of these plant parts might threaten the life of the mother plant.

Simple methods (crushing, powdering, and wetting) and equipment (stones, knives, and cups) were commonly employed, which might be because of inadequate training and processing equipment which are essential for drug formulation procedures. Additives and solvents of different types were commonly used for remedy preparation. These include honey, milk, sugar, injera/bread, water, and local alcohol, which are also vital for improving odor, solubility, and taste. Water comprised the highest percent for the preparation and administration of remedies. Because it has a high ability to extract different phytochemicals from plant origin. This finding agrees with the findings of other studies conducted elsewhere [11, 44–46].

As depicted in the figure, practitioners prefer the oral route as a common way of administration. This might be due to the relative easiness of this route, lack of ability to administer remedies by other parenteral routes, and fear of associated adverse effects if overdose occurs. This finding agrees with a study conducted in Dega Damot District [11], Tigray Region [18], Hawassa zuria district [38], Addis Ababa [47], Gemad District [48], and Kenya [49]; however, other studies conducted in Southern Ethiopia revealed that most medicinal plant preparations were administered cutaneously [50].

Taeniasis, ascariasis, amoebiasis, and giardiasis were the common gastrointestinal parasite infections treated by medicinal plants. Unspecified intestinal parasite infections were also reported. The action of these medicinal plants is supposed to be direct damage to the immature stages or adult worm expulsion. These plant extract products interfere with the central targets of intestinal parasites [2].

## 5. Conclusions and Recommendations

In this ethnobotanical review, a total of 85 medicinal plants have been identified and recorded for their use in the treatment of gastrointestinal parasitosis. The information obtained from this review can serve as a guide to discover novel antiparasitic agents from plants. Therefore, it is advisable for field researchers to properly identify, document, and conserve these medicinal plants and to conduct safety and efficacy studies on such claimed medicinal plants.

## Data Availability

The datasets used and/or analyzed during the current study are included in this article.

## Supplementary Materials

Supplementary Materials Table S1 supplement: summary of medicinal plants used for the treatment of intestinal parasitosis.
