# Supplementary material for: Medicinal Plants Utilized for the Treatment of Gastrointestinal Parasitosis in Ethiopia
Source: J Parasitol Res. 2022 Mar 16;2022:3584861. doi: 10.1155/2022/3584861 (PMC8942683; doi:10.1155/2022/3584861)
Supplement: Supplementary Materials — Table S1 supplement: summary of medicinal plants used for the treatment of intestinal parasitosis. [file 3584861.f1.docx]

**Table S1**: Summary of medicinal plants used for the treatment of intestinal parasitosis (Am= Amharic, Or=Oromifa, Tig=Tigrigna, Gu=Gumze, M=Maale A=Ari, Ku=Kunama, Daw=Dawro, Ged=Gedo, Si=Sidama, PU=parts used, RoA=Route of administration, IP=Intestinal parasitosis)

| S. no | **Scientific name** | **Family name** | **Local name** | **Habit** | **PU** | **Specific use** | **Method of preparation** | **RoA** | **References** |
| --- | --- | --- | --- | --- | --- | --- | --- | --- | --- |
| 1 | *Bersama abyssinica* Fresen. | Melianthaceae | Azamira (Am) | shrub | Leaf | Ascariasis | Crushing the leaf and drink it | Oral | [11] |
|  |  |  | Azamir (Am) | Shrub | Leaf | Ascariasis | Crush and powder, boil with tea then drink juice | Oral | [12] |
| 2 | *Hagenia abyssinica* (Bruce ) J.F.Gmel | Rosaceae | Soyid'uwa (Daw) | Tree | Fruit | Taeniasis | crushed and mixed with water taken | Oral | [13] |
|  |  |  | *Dadako* (Si) | Tree, | Leaf | Taeniasis | Decoction from berry and leaves | Oral | [14] |
|  |  |  | Kosso (Am) | Tree | Dried fruit | Taeniasis | Half cup of dried fruit powder | Oral | [15] |
|  |  |  | Kosso (Or) | Tree | Flower | Taeniasis | The flower is dried,  crushed, and boiled in water | Oral | [16] |
|  |  |  | ‘Kosso’ (Or) | Tree | Fruit | Ascariasis | Crushed dry fruits mix the powder with honey and a little bit of water and then boil and drink before breakfast for 5 days. | Oral | [17] |
|  |  |  | ‘Kosso’ (Or) | Tree | Fruit | Unspecified IP | Crushed dry fruit , mix the powder with local 'tella' and leave for overnight and drink before breakfast for three days | Oral |  |
|  |  |  | Koso (Am) | Tree | Leaf | Taeniasis | Crushing the fresh leaves and mix with water and drink it once | Oral | [11] |
|  |  |  | Habi (T) | Tree | Leaf, fruit  and  flower | Taeniasis | Crush, filter and drink the fluid alone or with milk | Oral | [18] |
|  |  |  | Kosso (Am)  Hetto (Or) | Tree | Fruit | Taeniasis | Dray flowers are pounded and resulting powder mixed with water. | Oral | [19] |
|  |  |  |  |  | Seed, Leaf | Unspecified IP | Mixture of leaf infusion and oil 1extracted from seed taken oral as anthelmintic | oral | [20] |
| 3 | *Sida schimperiana*  Hochst. ex A.Rich. | Malvaceae | Girawa (Am) | shrub | Leaf | Taeniasis | Crushed mixed with water | Oral | [21] |
|  |  |  |  |  |  | Ascariasis | Crushed mixed with water | Oral |  |
| 4 | *Vernonia*  *adoensis* Sch.  Bip. ex Walp. | Asteraceae | Eras abera/ Este musaye (Am) | Shrub | Root | Amoebiasis | powder then drink with water or Chew and swallow juice | Oral | [12] |
|  |  |  |  |  |  | Giardiasis | powder then drink with water or Chew and swallow juice | Oral |  |
| 5 | *Vernonia auriculifera* Hiern | Asteraceae | Reejii(Or) | Shrub | Stem | Amoebiasis | Peeling the young stem near the meristem and chewing it. | Oral | [22] |
| 6 | *Kleinia squarrosa* Cufod*.* | Asteraceae | Lulo (Am) | Shrub | Stem | Unspecified IP | Crush and taken oral as antihelminth | Oral | [20] |
| 7 | *Echinops kebericho* Mesfin | Asteraceae | Kebericho (Am) | Shrub | stem | Taeniasis | Drying and crushing then drink by mixing with *Capsicum annuum* L. and salt | oral | [11] |
| 8 | *Embelia schimperi* Vatke | Myrsinaceae | Enkoko (Am) | liana | Seed | Taeniasis | Crushing the seed and drink with alcohol | Oral |  |
|  |  |  | Hanquu (Or) | Liana | Fruit | Taeniasis | Fruit eaten early in the morning | Oral | [23] |
|  |  |  | Enkoko (Am) | liana | Fruit | Taeniasis | Crashed and drinking 1 cup juice | Oral | [24] |
|  |  |  | Enkoko (Am) | liana | Fruit | Taeniasis | Eat fresh or crush and  drink with ‘TELA DIFDIF’ | Oral | [12] |
|  |  |  | Haanquu(Or) | Liana | Seed | Taeniasis | Crushing the seeds, making s/n and drinking/ giving one water glass. Crushing the seeds, 208making s/n and drinking/ giving one water glass. | Oral | [22] |
|  |  |  | K'uank'uula(Daw) | liana | Fruit | Taeniasis | crushed mixed with water and taken orally before breakfast | Oral | [13] |
| 9 | *Combretum collinum* Fresen. | Nyctaginaceae | Babella (Ku)  Bissina (Tig) | Tree | Bulb | Taeniasis | Pounding, the fine powder is baked or cooked with powder of teff ” taffa Kitta” or “Kallafa’’ teff respectively and then eat or drink in the morning in empty stomach | Oral | [25] |
| 10 | *Glinus lotoides* L. var*lotoides* | Molluginaceae | Mataharree (Or) | Tree | Leafy-stem | Taeniasis | Leafy-stem of G. lotoides is crushed, powdered and liquefied. | Oral | [26] |
|  |  |  | Meterea (Am) | Herb | Seed | Taeniasis | Powder Mixed with water | Oral | [21] |
|  |  |  |  |  |  | Taeniasis | Powder paste | Oral |  |
|  |  |  |  |  | Leaf | Taeniasis | Powder mixed with water | Oral |  |
| 11 | *Brucea antidysentrica* J.F. Mill. | Simaroubaceae | Qomanyoo(Or) | Shrub | leaf | Ascariasis | Fresh leaves crushed and mixed with Leaves of *Bersema abyssinica* Fresen. and cooked With porridge and given for a person in need | Oral | [23] |
| 12 | *Caylusea abyssinica* (Fresen.) Fisch. &Mey. | Residaceae | Illancoo (Or) | Herb | Leaf | Amoebiasis | Fresh leaves cooked and eaten with ‘enjera’ /bread | Oral |  |
| 13 | *Calpurnia aurea* (Ait.) Benth. | Fabaceae | Ceekaa(Or) | Shrub | Leaf | Ascariasis | 9 juvenile leaves of Calpurnia aurea, 9 leaves *of Senna occidentalis* and 9 juvenile leaves of *Clausena anisata* smashed and the extracts taken. One cup of tea is given for man and half cup for Children | Oral |  |
|  |  |  | Hetsawets (Tig) | Shrub | Seed | Amoebiasis | Seeds are roasted on iron sheet, ground into powder, mixed with honey, dissolved in cup of water and taken for three days | Oral | [27] |
| 14 | *Securidica*  *longipedunculata* Fresen. | Polygalaceae, | Etsamanaay  (Or) | Shrub | Root | Unspecified IP | Root is pounded and mixed with H2O | Oral | [26] |
|  |  |  | Tsa-tse(Tig) | Shrub | Fruit | Taeniasis | The fruit is eaten or crushed and pounded and taken orally | Oral | [28] |
| 15 | *Myrsine africana* L. | Myrsinaceae | Kechema (Or) | Shrub | Fruit | Ascariasis | Fruit powder pasted with niger seed is eaten. | Oral | [16] |
|  |  |  |  |  |  | Taeniasis | Fruit powder pasted with niger seed is eaten. | Oral |  |
| 16 | *Caylusea abyssinica* (Fresen.)Fisch. & Mey | Resedaceae | Sheggitae(Gedo) | Herb | Root | Ascariasis | Crushed/pounded fresh/dry root water is taken orally | Oral | [29] |
| 17 | *Solanummariginatum* L.f. | Solanaceae | Aby ungule(Tig) | Shrub | Root | Ascariasis | Crush by mixing with roots of *Zehneria scabra*, and *Verbena officinalis*, filter and drink the fluid | Oral | [18] |
| 18 | *Coffea arabica* L. | Rubiaceae | Buna(Tig) | Shrub | Seed | Amoebiasis | Seeds are roasted, pounded into powder, mixed  with honey and taken orally | Oral | [27] |
| 19 | *Euphorbia cactus* Boiss. | Euphorbiaceae | Kolqualhamat(Tig) | Shrub | Latex | Ascariasis | Four drops of latex are mixed with sugar solution | Oral |  |
| 20 | *Jasminum grandiflorum* L. *subsp.floribundum* (R.Br. ex Fresen.) P.S. Green | Oleaceae | Habi-tselim (Tig) | Liana | Leaf | Ascariasis, | Leaves are crushed, squeezed and cup of juice with sugar is taken orally | Oral |  |
|  |  |  |  |  |  | Taeniasis | Leaves are crushed, squeezed and cup of juice  with sugar is taken orally | Oral |  |
| 21 | *Lepidium sativum* L. | Brassicaceae | Shenfa(Tig) | Herb | seed | Amoebiasis | Seeds are ground into powder, mixed with honey and then taken for three days | Topical |  |
|  |  |  | Feaxxo(Ged) | Herb | Seed | Unspecified IP | Dry seed powder is taken as with coffee as drink | Oral | [29] |
| 22 | *Premna oligotricha* Baker | Lamiaceae | Sasa hadima(Tig) | Shrub | Leaf | Ascariasis | Leaves are crushed and squeezed and a cup of juice is taken once orally | oral | [27] |
| 23 | *Linum usitatissimum* L. | Linaceae | Entatie(Tig) | Herb | Seed | Amoebiasis | Seeds are ground, mixed with water and a cup of juice drunk in the morning | Oral |  |
| 24 | *Olea europaea L subsp. Cuspidata*(Wall. ex G. Don) Cif. | Oleaceae | Awlie(Tig) | Tree | Leaf | Amoebiasis | Leaves are crushed, squeezed and a cup of taken orally | Oral |  |
|  |  |  |  |  |  | Ascariasis | Leaves are crushed, squeezed and a cup of juice taken orally for one day | Oral |  |
| 25 | *Oxalis anthelmintica* A. Rich | Oxalidaceae | Habachego (Tig) | Herb | Leaf | Taeniasis | Patient eats some and remains on diet for next three hours | Oral |  |
| 26 | *Ricinus communis* L. | Euphorbiacee | Gulie(Tig) | Shrub | Seed | Amoebiasis | Crushed seeds are mixed with water and taken with a cup of tea once | Oral |  |
| 27 | *Verbena officinalis* subsp.*africana*R. Fernandes & Verdc. | Verbenaceae | Atush(Tig) | Herb | Whole plant | Ascariasis | Plant is crushed, squeezed and juice taken with cup  of coffee for three days | Oral |  |
| 28 | *Otostegia*  *integrifolia* Benth. | Lamiaceae | Chiendog (Tig) | Shrub | Leaf | Ascariasis | Crush, filter and drink the fluid | Oral | [18] |
| 29 | *Justicia schimperiana* (Hochst.exA. Nees) T.Anders | Acanthaceae | Dummiuggae (Ged) | Shrub | Leaf | Unspecified IP | Pounded fresh/dry leaves are concocted with bark of *Croton macrostachyus* is taken orally for three days. | Oral | [29] |
|  |  |  |  |  |  | Giardiasis | Crushed, pounded fresh/dry leaf concocted with *Croton macrostachyus* in cold water is given as a drink for three days. | Oral |  |
|  |  |  | Sensel (Or) | Shrub | Leaf | Unspecified IP | Crushed fresh/dry leaves is concocted with bark of Croton macrostachyus is taken orally for three days | Oral | [17] |
|  |  |  | Smiza (Am) | Shrub | Leaf, stem | Taeniasis | Drink the concoction | Oral | [12] |
| 30 | *Kanahala laniflora* (Forssk.) R. Br. | Asclepidaceae | Wundiffo (Or) | Shrub | Root | Amoebiasis | Pounded fresh/dry root concocted with roots of *Croton macrostachys* and *Senna occidentalis* is taken orally | Oral | [29] |
|  |  |  |  |  |  | Giardiasis | Crushed fresh/dry root concocted with roots of *Croton macrostachys* and *Senna occidentalis* is taken orally | Oral | [17] |
| 31 | *Carduus leptacanthus* Fresen | Asteraceae | Guccino (Ged) | Herb | Stem | Ascariasis | Powdered dry stem mixed with butter is taken with coffee or tea. | Oral | [17,29] |
| 32 | *Carica papaya* L. | Caricaceae | Papaya(Ged) | Tree | Seed | Amoebiasis | Chewed and swallowed fresh seed | Oral | [29] |
|  |  |  |  |  |  | Unspecified IP | Chew and swallow seed | Oral |  |
| 33 | *Ensete ventricosum* (Welw.) Cheesman | Musaceae | Warqo(Ged) | Shrub | Root | Amoebiasis | Crushed/pounded fresh root with water is taken orally | Oral |  |
|  |  |  | ‘Warqe’(Or) | Shrub | Root | Amoebiasis | Crushed fresh root with water is taken orally | Oral | [17] |
| 34 | *Prunus africana* (Hook.F.) Kalkam | Rosaceae | T/kaka(Ged) | Tree | Root bark | Ascariasis | Crushed/pounded dry root bark mixed with water is taken as a drink | Oral | [29] |
|  |  |  | ‘Sukke’ (Or) | Tree | Root | Ascariasis | Crushed dry root bark mixed with water is taken as a drink | Oral | [17] |
| 35 | *Capsicum annuum.*L | Solanaceae | Miximixo (Ged) | Herb | Fruit | Ascariasis | Chew and swallow fresh/dry fruits | Oral | [29] |
| 36 | *Argemone mexicana* L*.* | Papaveraceae | Kossalae (Ged) | Herb | Leaf | Unspecified IP | Crushed and pounded fresh leaf mixed with leaf of *Vernonia amygdalina* is given orally. | Oral |  |
| 37 | *Aloe megalacantha*  Bark. | Aloaceae | Ere (Tig) | Shrub | Leaf | Amoebiasis | Crush leaves and squeeze juice, filter and drink | Oral | [18] |
|  |  |  |  |  | Latex | Ascariasis | Squeeze latex, filter and drink | Oral |  |
| 38 | *Euclea racemosa*  Murr.subsp. *schimperi* (A.DC.) F. White | Ebenaceae | Keleaw(Tig) | Shrub | Root | Amoebiasis | Remove bark of the root, boil it and drink the fluid with mancheba, milk product | Oral |  |
| 39 | *Euphorbiaabyssinica* J.F.Gmel. | Euphorbiaceae | Kulqual (Tig) | Tree | Latex | Ascariasis | Mix part with locally made beer and drink it or mix it enjera (local food) and eat it | Oral |  |
|  |  |  | Tekeze (Tig) | Tree | Root | Ascariasis | Chew and swallow the fluid | Oral |  |
| 40 | *Hypoestes forskaolii*  (Vahl) Roem. & Schult. | Acanthaceae | Girbia(Tig) | Herb | Root | Ascariasis | Boiling in milk with leaves of *Lantana trifolia* and drunk | Oral |  |
| 41 | *Ajuga integrifolia*Buch-Ham. | Lamiaceae | Endifdif (Tig) | Herb | Leaf | Ascariasis | Crush, filter and drink | Oral |  |
|  |  |  |  |  |  | Taeniasis | Crush, filter and drink | Oral |  |
| 42 | *Dovyalis abyssinica* (A. Rich.) Warb. | Flacourtiaceae | Mengolhats (Tig) | Shrub | Fruit | Amoebiasis | Eat the fruit or drink its juice | Oral |  |
|  |  |  |  |  |  | Taeniasis | Eat the fruit or drink its juice | Oral |  |
|  |  |  |  |  |  | Ascariasis | Eat the fruit or drink its juice | Oral |  |
| 43 | *Ficus vasta* Forssk. | Moraceae | Daero (Tig) | Tree | Bark | Ascariasis | Crush and it with honey | Oral |  |
| 44 | *Lantana trifolia* L. | Verbenaceae | Tsameo (Tig) | Shrub | Leaf | Ascariasis | Boil it with milk or tea and drink | Oral |  |
|  |  |  |  |  |  | Amoebiasis | Boil it in milk by mixing with roots of *Hypoestes forskaolii* and drink | Oral |  |
| 45 | *Merendra bengalensis* (Roxb) Benth. | Lamiaceae | Mesaguh (Tig) | Shrub | Leaf | Ascariasis | Crush, filter and drunk the fluid | Oral |  |
| 46 | *Oxalis corniculata* L. | Oxalidaceae | Chew Mirakut (Tig) | Herb | Bulb | Taeniasis | Peel the external part and eat it alone or mixed with enjera | Oral |  |
| 47 | *Rumex abyssinicus* Jacq. | Polygonaceae | Mequmeqo (Tig) | Herb | Leaf | Ascariasis | Crush, filter and drink the fluid | Oral |  |
|  |  |  | C'olieya (Daw) | Herb | Root | Ascariasis | decocted and half of a cup it taken | Oral | [13] |
| 48 | *Rumex nervosus* Vahl. | Polygonaceae | Hehot (Tig) | Shrub | Leaf& Stem | Ascariasis | Eat or chew and swallow the fluid | Oral | [18] |
| 49 | *Verbena officinalis* L. | Verbenaceae | Atush (Tig) | Herb | Root | Ascariasis | Crush it by mixing with roots of *Zehneria scabra*, filter and drink the fluid | Oral |  |
| 50 | *Zehneria scabra* (L.f.) Sond. | Cucurbitaceae | Hafaflo (Tig) | Climber | Root | Ascariasis | Crush by mixing it with *Verbena officinalis*, filter and drink the juice | Oral |  |
| 51 | *Coffee arabica* L. | Rubiaceae | Buna (Or, A) | Shrub | Fruit | Amoebiasis | The roasted fruit is powdered and liquefied with honey. | Oral | [16] |
| 52 | *Clausena anisata*  Hook. f. ex Benth | Rutaceae | Hulimay (Or) | Shrub(wild) | Leaf | Ascariasis | The decocted leaf is drunk. | Oral |  |
| 53 | *Cucurbita Pepo.* L. | Cucurbitaceae | Buqe (Or) | climber | Seed | Taeniasis | Seed is roasted and chewed. | Oral |  |
| 54 | *Carissa edulis* Vahl. | Apocynaceae | Agamsa (Or) | Shrub | Fruit | Ascariasis | Fruit is eaten | Oral |  |
| 55 | *Nigella Sativa* L. | Ranunculaceae | Tikur  Azmud (Am) | Herb | Fruit | Unspecified IP | -Powder mixed with honey | Oral | [19] |
| 56 | *Carica papaya* L. | Caricaceae | Papaya (Am) | Tree | Seed | Unspecified IP | Chewing of the seed | Oral |  |
| 57 | *Phytolocca*  *Dodecandar*L Herit. | Phytolaccaceae | Endod (Am) | Shrub | Root | Unspecified IP | Root, leaves and barks are  pounded and the concoction  is taken after drinking butter | Oral |  |
| 58 | *Brassica rapa*L. | Brassicaceae | Ejella (ku), Hamli adri (Tig) | Herb | Leaf | Taeniasis | Crushing the dried seed, the fine powder is homogenized with water and drink a cup of this solution. | Oral | [25] |
| 59 | *Citrus limon*L. Burm. f. | Rutaceae | Lemon (Tig) | Shrub | Leaf | Amoebiasis | Boiling the leaf with water, half cup of the decoction is taken orally in the morning in empty stomach | Oral |  |
| 60 | *Rumex nepalensis*  Spreng. | Polygonaceae | Tullet (A) | Herb | Leaf, root | Amoebiasis | Leaf and root powder is mixed with honey and taken. | Oral | [30] |
| 61 | *Ricins communis*L. | Euphorbiaceae | Mokkoniisaa(Or) | Tree | Bark, leaf | Taeniasis | Crushing the bark, boiling it and giving one coffee cup for | Oral  Topical | [22] |
| 62 | *Ehretia cymosa* Thonn. var.*cymosa* | Boraginaceae | Uraaga(Or) | Tree | Leaf | Amoebiasis | Crushing its leaves with the leaves of *Fagaropsis angolensis*, *Acmella caulirhiza* and internal part of stem bark of *Croton macrostachyus*, making s/n and drinking one glass water at once. | Oral |  |
| 63 | *Haplocoelum foliolosum*(Hiern) Bullock | Sapindaceae | Canaa(Or) | Shrub | Seed | Ascariasis | Chewing a handful of ripened seeds and swallowing it. | Oral |  |
| 64 | *Polyscias fulva* (Hiern) Harms | Araliaceae | Gudduba(Or) | Tree | Leaf | Amoebiasis | Chopping the leaves, making s/n and drinking one coffee cup. | Oral |  |
| 65 | *Maesa lanceolata* Forssk. | Myrsinaceae | Gegec'uwa (Daw) | Tree | Bark | Ascariasis, | crushed or powdered fresh mixed with water and taken | Oral | [13] |
| 66 | *Syzygium guineense* (Willd.) Dc. | Myrtaceae | Ocha (Daw) | Tree | Bark | Ascariasis, | crushed or powdered fresh mixed with water decocted and taken | Oral |  |
| 67 | *Rumex stetudelli* Hochst. ex A. Rich. | Polygonaceae | Tult (Am) | Herb | Root | Amoebiasis | Chewing | Oral | [21] |
| 68 | *Balanites aegyptiaca* (L.) Del. | Balanitaceae | Kulen (Am) | Tree | Seed | Unspecified IP | Pounded and mixed with food and eaten in empty stomach. | Oral | [31] |
| 69 | *Indigofera* sp. | Fabaceae | Mey dah  Dere (Or ) | Shrub | Leaf | Unspecified IP | Dry or fresh root crushed and mixed with water and taken oral. | Oral |  |
| 70 | *Halothamnus somalensis* (N. E. Br.) Botsch | Chenopodiaceae | Mirow(Am) | Shrub | Root | Unspecified IP | Dry or fresh root crushed and mixed with water and taken orally. | Oral |  |
| 71 | *Capparis cartilaginea* Decne. | Capparidaceae | Qelemberur(Or) | Tree | Root | Ascariasis | Dried root is crushed and boiled and consumed empty stomach. | Oral |  |
| 72 | *Azadirachta indica*  A. Juss. | Meliaceae | Kinina (Or) | Tree | Seed | Unspecified IP | Oil from seed Flower extra | Oral | [32] |
| 73 | *Cordia africana* Lam. | Boraginaceae | Kunjigsha (Ku), Awehi (Tig) | Tree | Fruit | Taeniasis | By eating the fresh fruit and swallow the seed | Oral | [25] |
| 74 | *Albizaanthelmintica* (A. Rich.) Brongn | Fabaceae | Bsana(Am) | Tree | Bark | Taeniasis | prepare by mixing with food | Oral | [33] |
| 75 | *Acanthus sennii* Chiov. | Acanthaceae | Key kusheshilie (Am) | Shrub | Root | Taeniasis | Pound, immerse in water then drink the juice | Oral | [12] |
| 76 | *Achyranthes aspera* L. | Amaranthaceae | Telenj (Am) | Herb | Root | Taeniasis | Crush, insert in water & drink | Oral |  |
| 77 | *Buddleja polystachya*  Fresen. | Loganiaceae | Anfar (Am) | shrub | Leaf | Unspecified IP | Crush and powder, immerse in  TEJ then drink the juice | Oral |  |
| 78 | *Celosia trigyna* L. | Amaranthacea | Lemlemcho (Am) | Herb | Seed | Taeniasis | Grind and drink with water | Oral |  |
| 79 | *Croton macrostachyus* Del. | Euphorbiaceae | Misana (Am) | Tree | bulb | Taeniasis | Crush, pound, then drink  Juice | Oral |  |
|  |  |  |  |  | Leaf | Taeniasis | Boil, grind, make it WOTE (souse) with butter then eat with ENJERA | Oral |  |
|  |  |  | Bissana (Am) | Tree | Bulb | Taeniasis | Powder Mixed with water | Oral | [21] |
| 80 | *Cynodon dactylon* (L.)  Pers. | Poaceae | Serdo (Am) | Herb | Leaf, stem | Taeniasis | Drink the concoction | Oral | [12] |
| 81 | *Dodonaea angustifolia* L.f. | Sapindaceae | Kitkita (Am) | Shrub | Root, leaf | Taeniasis | Pound, immerse in water and drink the diluted mixture | Oral |  |
|  |  |  |  |  | Leaf, stem | Taeniasis | Drink the concoction | Oral |  |
|  |  |  | Edecha (Or) | Shrub | Leaf | Unspecified IP | Fresh leaf extract taken oral as anthelminthics | Oral | [20] |
|  |  |  | Edechaa (Or) | Shrub | Root | Unspecified IP | Rubbed | Anal | [32] |
| 82 | *Lactuca intermis*  Forssk. | Asteraceae | Dememerarit (Am) | Herb | Root | Amoebiasis | Chew and swallow the juice | Oral | [12] |
| 83 | *Laggera crispata* (Vahl) Hepper & Wood | Asteraceae | Keskesso/ alshasume (Am) | Herb | Leaf , | Taeniasis | Crush and drink with water | Oral |  |
| 84 | *Prunuspersica* (L.) Batsch | Rosaceae | Kok (Am) | Shrub | Leaf, stem | Taeniasis | Drink the concoction | Oral |  |
| 85 | *Withania somnifera* (L.) Dunal in DC. | Solanaceae | Giziewa (Am) | Herb | leaf | Taeniasis | Fumigate in a closed fashion | Topical |  |
